# Supplementary material for: Causal Relationship between Aging and Anorexia Nervosa: A White-Matter-Microstructure-Mediated Mendelian Randomization Analysis
Source: Biomedicines. 2024 Aug 16;12(8):1874. doi: 10.3390/biomedicines12081874 (PMC11351342; doi:10.3390/biomedicines12081874)
Supplement: Supplementary file 1 [file biomedicines-12-01874-s001.zip › Supplementary Figure/Figure S4/Singe SNP plot of MR analysis for TL to ubm-b-1484.pdf]

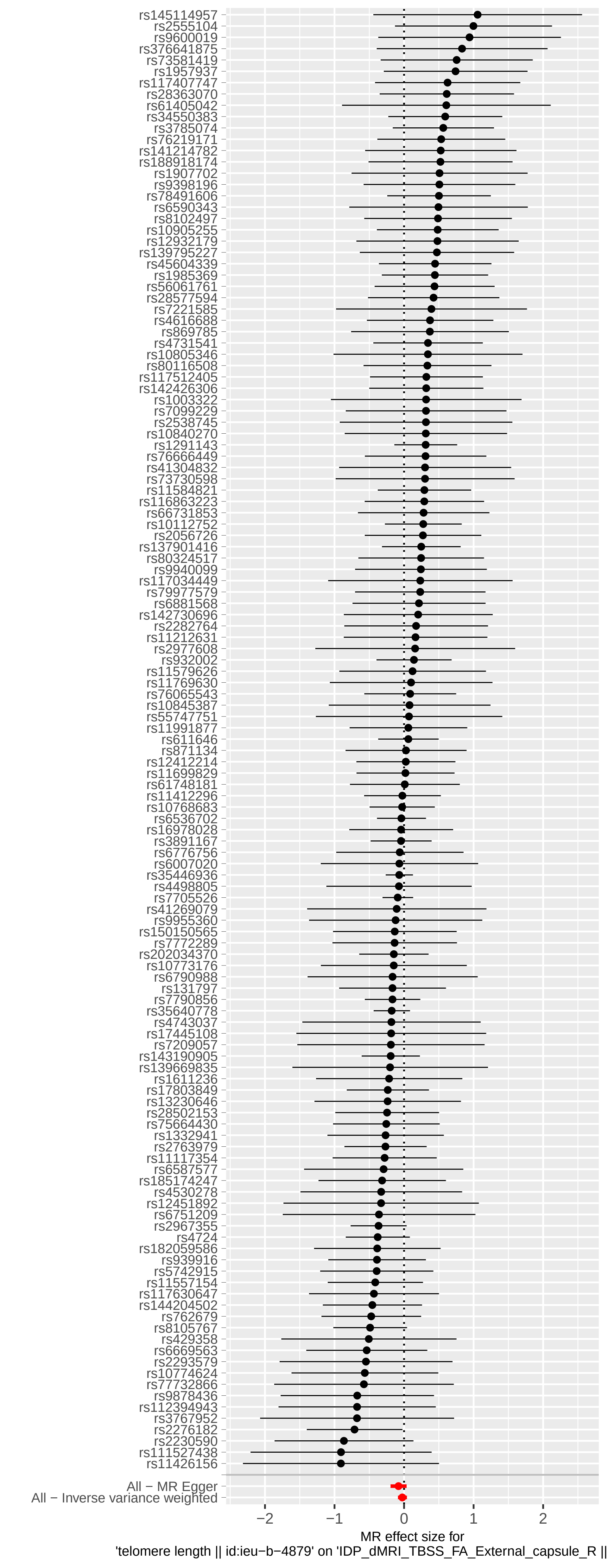
rs145114957
rs2555104
rs9600019
rs376641875
rs73581419
rs1957937
rs117407747
rs28363070
rs61405042
rs34550383
rs3785074
rs76219171
rs141214782
rs188918174
rs1907702
rs9398196
rs78491606
rs6590343
rs8102497
rs10905255
rs12932179
rs139795227
rs45604339
rs1985369
rs56061761
rs28577594
rs7221585
rs4616688
rs869785
rs4731541
rs10805346
rs80116508
rs117512405
rs142426306
rs1003322
rs7099229
rs2538745
rs10840270
rs1291143
rs76666449
rs41304832
rs73730598
rs11584821
rs116863223
rs66731853
rs10112752
rs2056726
rs137901416
rs80324517
rs9940099
rs117034449
rs79977579
rs6881568
rs142730696
rs2282764
rs11212631
rs2977608
rs932002
rs11579626
rs11769630
rs76065543
rs10845387
rs55747751
rs11991877
rs611646
rs871134
rs12412214
rs11699829
rs61748181
rs11412296
rs10768683
rs6536702
rs16978028
rs3891167
rs6776756
rs6007020
rs35446936
rs4498805
rs7705526
rs41269079
rs9955360
rs150150565
rs7772289
rs202034370
rs10773176
rs6790988
rs131797
rs7790856
rs35640778
rs4743037
rs17445108
rs7209057
rs143190905
rs139669835
rs1611236
rs17803849
rs13230646
rs28502153
rs75664430
rs1332941
rs2763979
rs11117354
rs6587577
rs185174247
rs4530278
rs12451892
rs6751209
rs2967355
rs4724
rs182059586
rs939916
rs5742915
rs11557154
rs117630647
rs144204502
rs762679
rs8105767
rs429358
rs6669563
rs2293579
rs10774624
rs77732866
rs9878436
rs112394943
rs3767952
rs2276182
rs2230590
rs111527438
rs11426156
All − MR Egger
All − Inverse variance weighted
−2
−1
0
1
2
MR effect size for
'telomere length || id:ieu−b−4879' on 'IDP_dMRI_TBSS_FA_External_capsule_R ||
